# Calibration Uncertainty of Non-Catching Precipitation Gauges

**DOI:** 10.3390/s22176413

**Published:** 2022-08-25

**Authors:** Quentin Baire, Miruna Dobre, Anne-Sophie Piette, Luca Lanza, Arianna Cauteruccio, Enrico Chinchella, Andrea Merlone, Henrik Kjeldsen, Jan Nielsen, Peter Friis Østergaard, Marina Parrondo, Carmen Garcia Izquierdo

**Affiliations:** 1 National Standards, FPS Economy, 16 Boulevard du Roi Albert II, B-1000 Brussels, Belgium; 2 Department of Civil, Chemical and Environmental Engineering, University of Genova, 16145 Genova, Italy; 3 WMO Lead Centre “B. Castelli” on Precipitation Intensity, 16145 Genova, Italy; 4 Applied Thermodynamics Program, Istituto Nazionale di Ricerca Metrologica, 10135 Torino, Italy; 5 Division of Energy & Climate, Danish Technological Institute, 8000 Aarhus, Denmark; 6 Centro Español de Metrología, 28760 Tres Cantos, Spain

**Keywords:** precipitation, measurement, calibration, uncertainty, non-catching gauges

## Abstract

Precipitation is among the most important meteorological variables for, e.g., meteorological, hydrological, water management and climate studies. In recent years, non-catching precipitation gauges are increasingly adopted in meteorological networks. Despite such growing diffusion, calibration procedures and associated uncertainty budget are not yet standardized or prescribed in best practice documents and standards. This paper reports a metrological study aimed at proposing calibration procedures and completing the uncertainty budgets, to make non-catching precipitation gauge measurements traceable to primary standards. The study is based on the preliminary characterization of different rain drop generators, specifically developed for the investigation. Characterization of different models of non-catching rain gauges is also included.

## 1. Introduction

Comparability of meteorological observations, among different networks and instruments, is fundamental to improve forecasting modeling and understanding of the climate evolution. To achieve robust comparability, data traceability [1] to recognized standards is needed and is provided by instrument calibration [2]. Climate studies, as well as hydrological, meteorological, and agricultural applications—among others—rely on instruments which measure liquid/solid atmospheric precipitation.

Correct data interpretation and meaningful comparison among different stations, as well as among different systems and methods from various regions and different operational networks, can only be performed when a common ground for evaluating the measurement uncertainty is provided. Non-catching precipitation gauges are increasingly being adopted in operational networks, thanks to a number of advantages over more common catching-type gauges, especially for Automatic Weather Stations. CEN TC318 “Hydrometry” has identified specific testing and measurement needs for non-catching instruments to measure liquid/solid atmospheric precipitation.

At present, however, standard calibration procedures for such kind of instruments are missing as well as the associated uncertainty budgets. Manufacturers of meteorological instruments are developing non-catching gauges to meet the requirements of national meteorological and hydrological services and are facing calibration issues due to the nature of the measurand and the highly varying microphysical characteristics of precipitation, even within the same event. Today, each manufacturer implements its own calibration system, either simulating the falling drops with metallic or glass spheres or using simplified raindrop generators [3]. Traceability of such procedures is still a challenge. To support reliable use of these instruments, and as a development beyond the recent standard adopted under CEN TC318 for the calibration of catching-type gauges, traceable instrument calibration methods for non-catching gauges used to measure precipitation amount and intensity have been identified as a requirement.

In response to these needs, the work presented here is based on a metrological approach to establish measurement traceability for non-catching precipitation gauges, by studying, testing, and proposing specific calibration procedures. Such procedures consider the working principles of the devices under test and involve different models of drop generators, specifically developed for the study. The uncertainty budget for the calibration is, therefore, structured in three groups of components: the generated drops dimensions and frequency, the environmental factors, and the main properties of the instrument under calibration. Organizing the uncertainty in separate groups of components allows the user to fix some parameters during the calibration, making the overall uncertainty evaluation easier.

Such process is also aligned to the “Measurement Quality Classification Scheme (MQCS)” [4], recently approved by the Commission on Infrastructure of the World Meteorological Organization (INFCOM-WMO) and included in the WMO Guide on Instruments and Methods of Observation (WMO-No. 8) [5]. The MQCS prescribes to divide uncertainties in separate groups of components, in order to better identify those contributions due to the measuring system, the calibration, the environmental effects (also defined as associated quantities of influence), up to the maintenance schedule, the system coupling and, even more separated, the siting characteristics.

The identification of measurement uncertainty, as investigated here, is part of the growing contribution brought by the metrological community to the environmental observations, in meteorology and climatology. Joint research projects, such as the group of projects “MeteoMet—Metrology for Meteorology” [6,7], recently delivered support in understanding, evaluating, and reducing uncertainties. These projects addressed questions such as data quality through documented traceability, improved and dedicated calibration procedures, specific studies on instrument characteristics and comparison of different systems, but also development of novel instrumentation for both laboratory and field calibration. These topics were also discussed at dedicated events [8].

The relevance of traceability in precipitation records, to achieve reference-grade data quality is also well identified by the Global Climate Observing System (GCOS) in establishing the new “Surface Reference Network (GSRN)” [9].

The work reported here was carried out as part of the “MeteoMet” initiatives in the framework of a specific joint research project titled “Calibration and accuracy of non-catching instruments to measure liquid/solid atmospheric precipitation (INCIPIT)”, funded in 2019 by the European Metrology Programme for Research and Innovation (EMPIR) of the EURAMET, the association of European National Institutes of Metrology. This project established a sound metrological basis for the evaluation of the calibration uncertainty, detecting and analyzing the relevant sources of uncertainty in the corresponding calibration procedures.

The result of this study will be organized in terms of a normative proposal. A report was prepared by the EURAMET “INCIPIT” project partners to act as a proposal for a normative document to be discussed at CEN TC318 “Hydrometry”, WG12 “Rainfall Intensity”, following the approval within that TC on February 2022 of a new work item (NWI) for a European norm entitled “Calibration and accuracy of non-catching precipitation measurement instruments”. A second document, based on the previous deliverables of the project, was prepared, and submitted to the same CEN TC318, following the approval within that TC on February 2022 of a new work item (NWI) for a Technical Report entitled “Metrological requirements and test methods for non-catching-type rain gauges”.

## 2. Uncertainty Model

The modeling approach proposed in this work is based on the work of Sommer et al. ([10,11]), depicting the cause-and-effect relationships of the measurements to be analyzed and modeled as a block diagram. In [10], three standard blocks are described:

1. Parameter sources (SRC): providing measurable quantity.
2. Transmission units (TRANS): any kind of signal processing and influencing.
3. Indicating units (IND): indicate input quantities.

**Figure 1:**
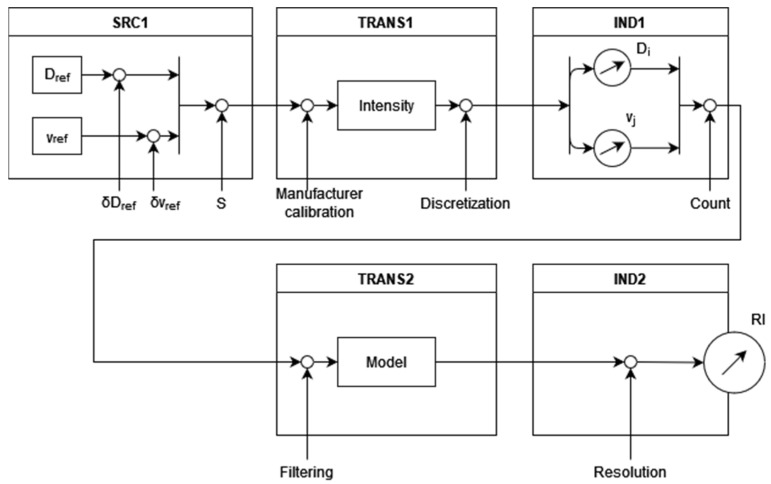
Uncertainty model for non-catching precipitation gauges.

The model proposed here, and presented in Figure 1, is a two-staged model. The instruments studied here use drops as measurable quantities, transformed into either Particle Size Distribution (PSD) or Particle Velocity and Size Distribution (PVSD)—depending on the working principle of the instrument. Some instruments provide values for those distributions, others do not. Either way, values of sizes (and velocities) are then used by the instrument to compute the Rainfall Intensity. Therefore, the first IND block, IND1 in Figure 1, is also a parameter source for the indication of rain intensity given by the instrument.

Our measurand is the Rainfall Intensity (RI), i.e., the volumetric flow of the liquid portion of atmospheric precipitation. According to the Guide to Meteorological Instruments and Methods of Observation [5] published by the World Meteorological Organization (WMO), precipitation is defined as “the liquid or solid products of the condensation of water vapor falling from clouds or deposited from air onto the ground”, and precipitation intensity is defined as “the amount of precipitation collected per unit time interval. The total amount of precipitation which reaches the ground in a stated period is expressed in terms of the vertical depth of water (or water equivalent in the case of solid forms) to which it would cover a horizontal projection of the Earth surface”.

The Rainfall Intensity measured in mm/h by the instrument can be obtained as:

(1) $$R=\frac{4}{3}\pi \sum_{i,j}\left(\frac{1}{S}\frac{N_{i,j}}{\Delta t}{\left(\frac{D_{i}}{2}\right)}^{3}\right),$$

where S is the detection surface of the instrument, ∆*t* the sampling interval, *N_i_*_,*j*_ is the PVSD (we will use *N_i_* for devices providing only a PSD) and $D_{i}$ the drop diameter. The index i is for the ith class of diameter, while the index j is for the jth class of velocity in the distribution. In this study, if provided by the device under test, we will use the diameter and velocity as the measurand. With Equation (1), this information is sufficient to retrieve the RI, and avoid any algorithm-related effect that might occur between this value and the indication of the RI by the instrument. This is equivalent as ignoring the second stage of our block model for uncertainty.

## 3. Uncertainty Block SRC1: Drop Generators

The measurable quantities for the non-catching rain gauges are the drop size and velocity. To produce reference drops in a controlled manner, three drop generators were developed within the INCIPIT project. The first drop generator, hereinafter named DG1, was developed by the Teknologisk Institut (DTI) in Denmark, the second one, DG2, was developed by the University of Genova (UNIGE) in Italy and the third one, DG3, by the FPS Economy, Metrology, National Standards (SMD) in Belgium.

The characterization of the drop generators provides the reference drop size and velocity (D_ref_ and v_ref_) in a traceable way, along with their respective uncertainties (δD_ref_ and δv_ref_). The descriptions of the three drop generators and their characterization are reported in the following subsections.

### 3.1. DTI Generator—DG1

The drop generator design is based on the following components: (1) a precision syringe pump, (2) a nozzle/needle and (3) a piezo-electric buzzer.

The setup consists of a syringe pump and a drop-generation chamber, the latter being equipped with a piezo-electric deflector membrane (“buzzer”) and a tube/nozzle. The syringe pump used is a TECAN syringe pump of the type CENTRIS. One key feature of this type of pump is that it is volumetric, i.e., it delivers a well-determined flow of liquid. The initial volume of the drops can be determined from the flow rate, *Q*, and the time interval between the drops, Δ*t*, by

(2) $$V_{drop}=Q\Delta t$$

**Figure 2:**
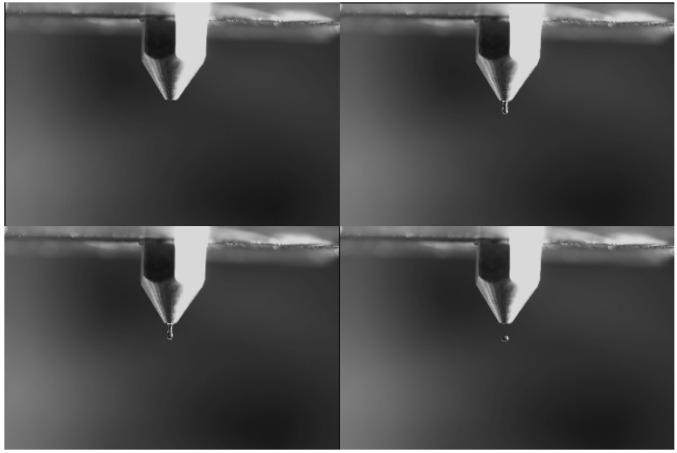
Formation of a small drop from the nozzle in four steps. Step 1 (**top left**): before the piezo pulse; Step 2 (**top right**): the water is being ejected by the piezo pulse; Step 3 (**bottom left**): due to inverse pulse the connection with the drop is starting to break apart; Step 4 (**bottom right**): the drop is now free and being further accelerated by gravity.

The drop diameters, given in Table 1, are all calculated under the assumption of spherical geometry. The formation of small drops, <1 mm, is similar to the work presented by Harris et al. in [12], and illustrated in Figure 2; the electric membrane pushes a thin meniscus of water out through the nozzle. The voltage on the electronic membrane is reversed, and the water is retracted into the water reservoir. Due to the inertia of the water outside the nozzle the meniscus separates from the nozzle, and a drop is generated. For the medium size drops, between 1 and 4 mm, the drops are produced with a known volume of water, controlled by the pump, and formed at the tip of the nozzle. The electric membrane is used to create a pressure pulse, which releases the drop from the nozzle. Large drops are produced as free-falling drops using a specially designed nozzle.

The creation of small- and medium-sized drops is verified via photo analysis, where a camera detects that exactly one drop is released for each pulse. Similarly, photo analysis was used to verify the velocity of the drops right above the rain gauge, in a procedure similar to the one developed by UNIGE to analyze their drop velocity (see Section 3.2).

When using different size nozzles/needles, the full range of drop sizes, shown in Table 1, can be generated.

### Uncertainty of Drop Volume

As the volumes of the drops are linearly dependent on flow rate and on time between drops, the uncertainty of the drops, when generated, can be found through a classical GUM analysis [2].

The flow is evaluated to have an uncertainty of 0.5%, while Δ*t* has an uncertainty of roughly 4 µs. It is thus negligible compared to the uncertainty of the flow at any reasonable Δ*t*.

Evaporation was analyzed theoretically and was found to be negligible (<0.05%).

Due to the above, the only significant uncertainty component is the flow rate, giving an uncertainty of 0.5% on the drop volume.

### 3.2. UNIGE Generator—DG2

**Figure 3:**
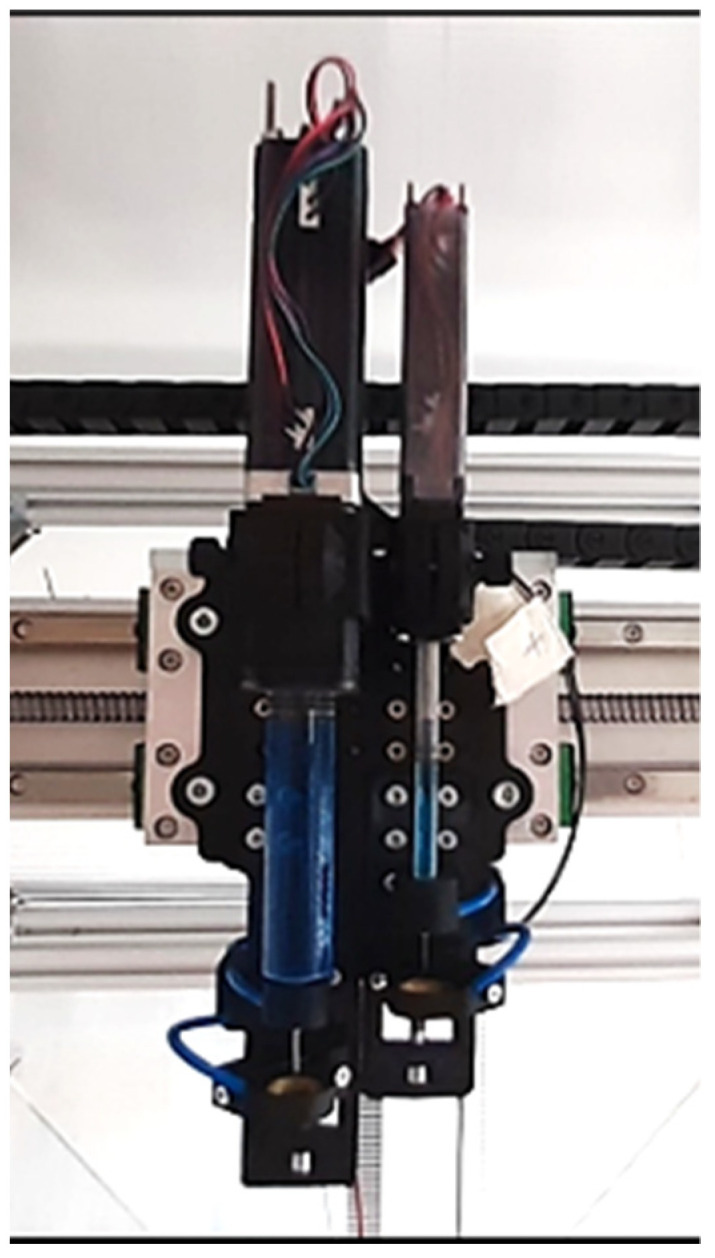
DG2—double-syringe device for drop formation and detachment.

Two high-precision syringe pumps, with a capacity of 20 and 1 mL (piston diameter of 20 and 4.5 mm, respectively), are used to produce water drops of the required volume (see Figure 3). An electric field, generated by a high voltage trigger, allows releasing each single drop on demand. Each drop is generated at the tip of a suitable nozzle by dispensing the necessary volume to achieve the desired drop size and then detached by exploiting a 5 kV potential difference, where the water is negatively charged and attracted by a metal ring (positively charged), positioned just below the tip of the nozzle. By using different nozzles/needles and the proper syringe pump, drops of various sizes are produced.

**Figure 4:**
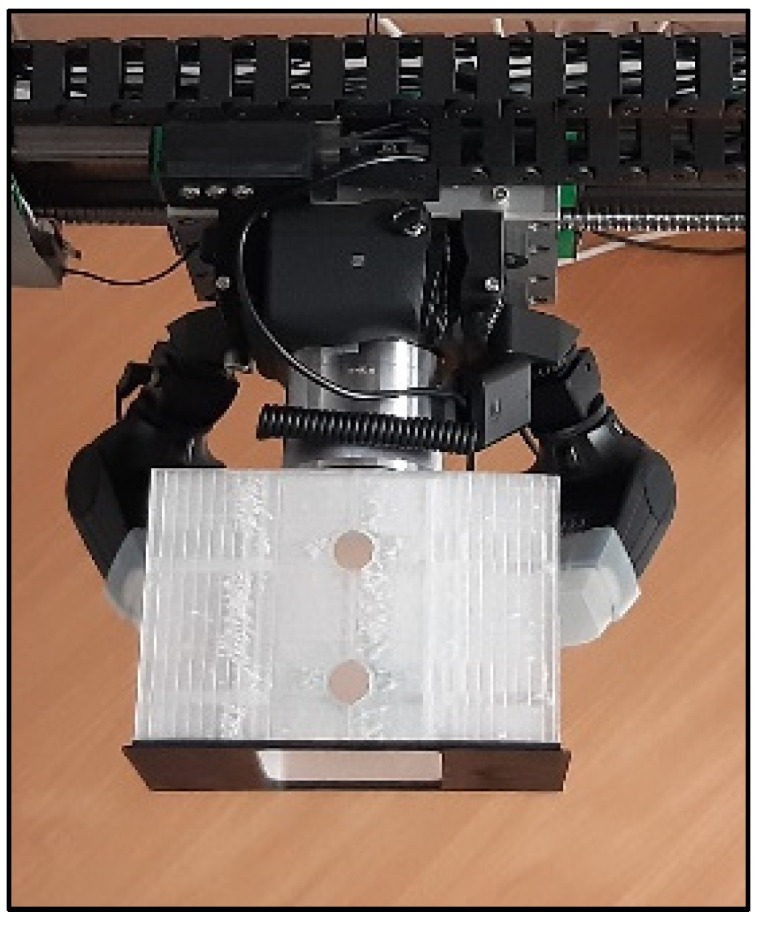
DG2—photogrammetric device for the verification of the generated drop size and velocity.

**Figure 5:**
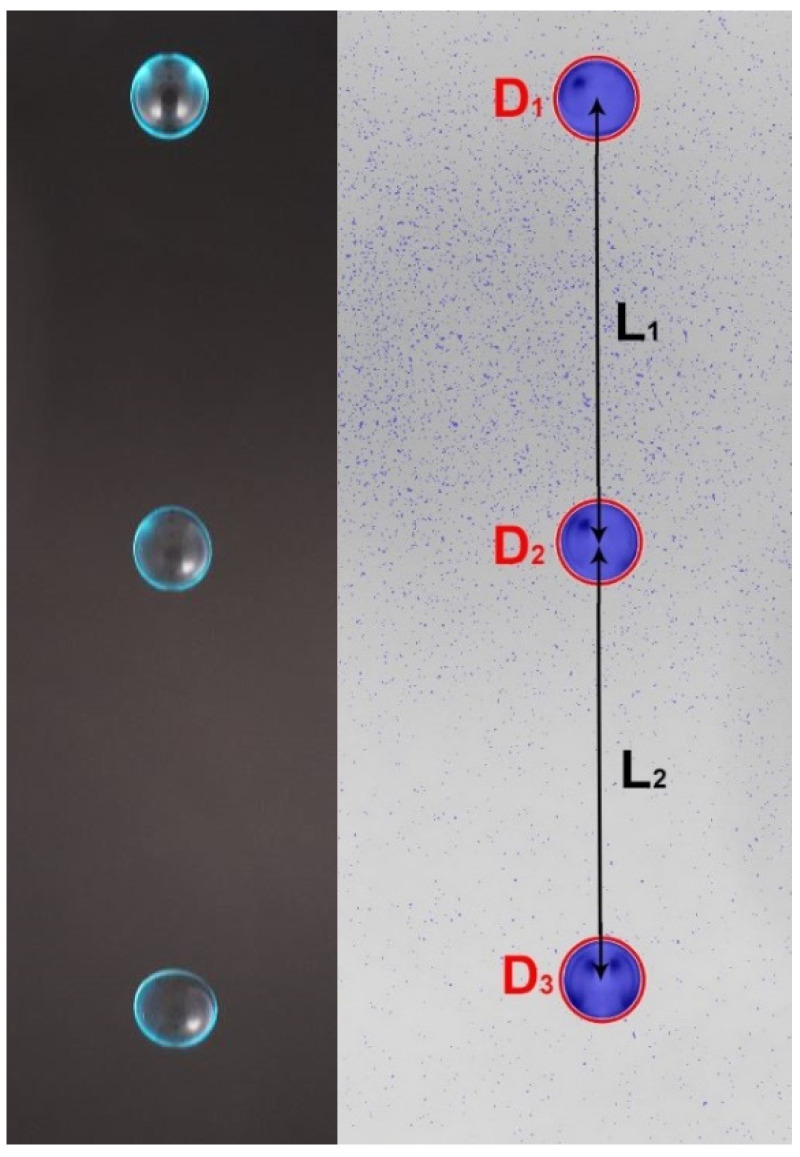
DG2—sample image of a single water drop in flight as released by the DG2 and captured three times in the same picture by the photogrammetric device (**left-hand side**). In the right-hand panel the same image is reported, after software processing elaboration, to show the equivolumetric circular shape of the drop (**D1**–**D3**, in red) and traveled distances (**L1** and **L2**).

To verify the size and fall velocity of the generated drops just above the sensing area of the instrument under test, a photogrammetric device is included in the drop generator assembly (see Figure 4). The system uses a high-resolution camera (Sony a6100) equipped with two flashes, which are triggered three times in a very short sequence (at 4.2 millisecond intervals) to capture three images of each drop in flight within a single picture (see Figure 5). The timing for the activation of the speedlights and the opening of the camera shutter are defined based on a numerical model of the drop vertical acceleration in still air [13].

Each image is processed by a dedicated software to derive the drop size (equivolumetric diameter) and fall velocity. An example of the processed images is reported in the right-hand side of Figure 5, where a single drop in three different positions is shown with the automatically detected drop circular contour (in red). The average drop diameter and fall velocity are equal to 3.474 mm and 4.372 m/s, respectively.

The photogrammetric method is traceable to the international standards of length and time. The time interval is obtained as a differential measure using the internal clock of the acquisition system that monitors the output of a photodiode. As for length measurements, three parameters are necessary: the pixel physical size (pixel pitch, *Pp*), the lens focal length (*Fl*) and the shooting distance (*Sd*). The sensor pixel pitch is provided by the manufacturer with high accuracy. The lens focal length can be measured and certified in optical laboratories. The shooting distance is directly measured on the device once assembled. The equivalent length of one pixel in the captured image (*Ip*) is obtained as:

(3) $$Ip=\frac{\left(Sd-Fl\right)}{Fl}\;\cdot \;Pp,$$

The uncertainty associated with the definition of *Fl* and *Pp* is assumed negligible compared to the uncertainty of *Sd* and the latter is mainly determined by the drop releasing mechanism. It was calculated from the observed drop positions in several images, assuming that the uncertainty of the drop position is isotropic. The resulting standard deviation for the shooting distance *Sd* ranges between 1.87 and 0.67 mm when the drop size is between 1.5 and 5 mm, respectively. These values lead to an uncertainty between ±2.4 × 10^−3^ mm and ±8.7 × 10^−3^ mm for *Ip*, depending on the drop size. The uncertainty on the photogrammetric determination of the drop size has a resulting maximum value equal to 0.016 mm when the drop size is equal to 1.5 mm.

Validation of drop size measurements was obtained by weighing the total volume of samples of about 20–50 drops with a precision balance having a resolution of 0.001 g. Drops were released at 1.20 m above the center of the measurement plane of the camera.

Results are summarized in Table 2 in terms of the average drop diameter obtained from the software and the balance, and their difference. This difference increases with the drop size since the photogrammetric detection overestimates the drop diameter when drops present an oblate section due to significant oscillations in their shape. This is due to those drops failing to approach the aerodynamic equilibrium during the flight, i.e., their terminal fall velocity (see the last column of Table 2).

A second set of tests was conducted using the photogrammetric system alone, without weighing the overall water volume. Statistics of the detected drop diameter and fall velocity are listed in Table 3 and Table 4 together with the number of released drops during each test.

The repeatability of the drop size is quite good, and the deviations of the maximum and minimum generated drop size from the average diameter are about equal to 110% and 90%, respectively. Also good is the consistency of the measured fall velocities and their repeatability. It is evident that the fraction of the terminal velocity that can be achieved with the fall height adopted in the tests (1.20 m) is about 90 % for the smallest drops (D = 0.85 mm), while it only reaches about 50 % for the largest drops (D = 3.42 mm).

Note that—although the good repeatability is a positive feature of the developed drop generator—using an embedded photogrammetric device to measure the actual size and velocity of each single drop as released by the system makes the strict repeatability of the drop characteristics a much less relevant issue. The measured diameter rather than the nominal one is indeed used for comparison with the diameter reported by the instrument under test. Analogously, the measurement of the fall velocity of each released drop avoids resorting to theoretical formulations and the need to adopt very tall supporting structures to achieve the terminal velocity of the largest drops.

### 3.3. SMD Generator—DG3

DG3 uses three peristaltic pumps and different nozzle geometries to generate drops of different sizes. The nozzle is mounted on a moving metallic structure in order to allow easy change of the drop position inside the measurement area. The displacement of the nozzle is controlled by two motors (stepper motor and servo motor).

Each nozzle was tested using a precise weighing instrument in order to have information on the drop sizes. Assuming all drops are equal, by weighting 100 drops 10 times for each nozzle, we estimated the uncertainty on the drop weight, see Table 5. This is propagated to the drop size by taking also into account the uncertainty contribution to the density of water at the room temperature.

We observed that the repeatability of the measurement is quite good but tends to be better for the smaller sizes. The characterization of the different nozzles shows that the probability density function (pdf) used here must be a normal distribution since the effects not modeled coming from the nozzle shape will affect the repeatability of the measurement.

Since this drop generator was built for test purposes, there were no velocity measurements. It can thus be used only for estimation of the drop size or be used at heights where the drop terminal velocity can be reached.

### 3.4. Other Contributions to Uncertainty Related to Reference Drop Diameters—$\delta D_{ref}$

The value of δD_ref_ in the uncertainty budget will come from the characterized drop used in the calibration process. If the unit under test is based on the determination of the size of the drop with an optical method (see Section 4), the shape of the drop will also affect this value.

For this type of instrument, the drops are supposed to be spherical. However, the raindrop shape can be defined in terms of the axial ratio (*b*/*a*) between the vertical (*b*) and the horizontal axis (*a*) [14]. The polynomial mathematical model, reflecting the characteristic flattened base that increases with drop size, is the following [15]:

(4) $$\frac{b}{a}=1.0048+5.7\;10^{-4}\;D-2.628\;10^{-2}D^{2}+3.682\;10^{-3}D^{3}-1.677\;10^{-4}D^{4},$$

Optical measurement principles exploited by non-catching-type gauge detect the dimension of the horizontal axis of the droplet to calculate its volume. For those gauges, the uncertainty introduced by this calculation will contribute to δD_ref_.

### 3.5. Contribution of the Detection Surface S

In Equation (1), the values of the detection surface *S* are those given by the manufacturer of the different devices. The literature shows that some effects might occur, depending on the type of instrument used, for instance in [16] for impact instruments or [17] for optical transmission instruments, influencing the determination of the size of the drop as a function of the location where it is detected.

For instance, for optical transmission instruments, DeMoraes et al. explain in [17] that the power distribution on the laser is not constant on the whole surface. This has an influence on the size determined by the instrument since for the same drop, the intensity reduction will be less important if it falls further from the center of the detection surface.

## 4. Uncertainty Block TRANS1: Non-Catching Disdrometer Models

Instruments that quantify rainfall (i.e., the flux of the drop size distribution at the surface) measure different properties of the flux, depending on the type of instrument [18].

Non-catching gauges count the number of particles that hit/cross their sensing surface/volume and measure raindrop characteristics such as dimension (drop diameter, D), fall velocity, and/or kinetic energy. From those data, the PSD or PVSD, precipitation intensity and other integral variables can be calculated. In our uncertainty modeling, this corresponds to signal processing, transforming the signal from the reference drop into a reading of the value for its size (and velocity).

A survey of the existing models of non-catching gauges (some of them named disdrometers) and an accurate description of their working principles is available [3]. Some details on how the size of the drop is determined are summarized below. 

### 4.1. Impact Disdrometers

Impact disdrometers determine the size of hydrometeors based on their kinetic energy when impacting the instrument. They can be divided in two categories: acoustic devices and displacement devices. The two types of instruments give PSD, the velocity being estimated by models.

Acoustic disdrometers are based on the piezoelectric effect. In those instruments, the voltage output U is proportional to the acting force, F, to the piezoelectric constant d and to the capacity of the sensor element, $C_{S}$.

To consider the total impact process, as described in [16], it is the integral over time of the output voltage that is considered. This is proportional to the momentum p(D) due to the force transferred by each impacting drop, basically the mass m of the drop times its velocity v, which can be written for a single drop before its impact as:

(5) $$\begin{array}{c} p\left(D\right)=mv=\frac{\pi }{6}\rho D^{3}\sqrt{{\left(v_{t}+v_{z}\right)}^{2}+v_{hor}^{2}}\; \end{array}$$

where ρ is the density of the fluid, $v_{t}$ the terminal velocity, $v_{z}$ the up or downdraft, $v_{hor}$ the horizontal velocity.

Combined with the previous description, the size of the hydrometeor can be written as a function of the integral over time of the output voltage as:

(6) $$\begin{array}{c} D=\sqrt[{3}]{\frac{C_{s}}{d}\frac{6}{\pi \rho }\frac{\int^{\;}Udt}{\sqrt{{\left(v_{t}+v_{z}\right)}^{2}+v_{hor}^{2}}}}\; \end{array}$$

### 4.2. Optical Disdrometers

Non-catching instruments based on the optical transmission measure the PVSD of hydrometeors. Based on the reduction in the intensity of a laser light sheet due to the shadow cast by each crossing hydrometeor and the duration of this shadow, a suitable measure of its diameter and fall velocity is obtained.

The basic measurement principle of these instruments is a reduction in the transmitted radiation $I_{0}$ due to each drop passing through the laser beam. The received intensity I is proportional to the size of the drop D and S, the vertical cross section of the light sheet. 

We can thus write D, the diameter of the hydrometeor, as

(7) $$\begin{array}{c} D=\sqrt{\frac{4}{\pi }\frac{I\;S}{I_{0}}} \end{array}$$

For the velocity, as described in [19], the computation is based on the integral of the signal over each raindrop pulse. This integral is proportional to $\frac{D^{2}}{v}$.

Optical scattering Present Weather Sensors also allow measurement of the PVSD of the hydrometeors, but with a different working principle. While a particle crosses a series of interference fringes generated by the transmitter, it measures the intensity of the scattered light at each detector. The velocity is derived from the time separation between pulses in the scattered intensity as the particle crosses two or more light sheets. The particle size is derived from relative time delays between signals received at different scattering angles, or by the phase measurements.

For an instrument where the light sheets are separated from a distance s, the velocity can be estimated using the time delay at detector A, $\Delta t_{A}$, or equivalently at detector B, $\Delta t_{B}$:

(8) $$\begin{array}{c} v=\frac{s}{\Delta t_{A}}=\frac{s}{\Delta t_{B}}\; \end{array}$$

Moreover, the configuration of the detector is such that, in practice, the detector A receives the signal before the detector B. The corresponding time delay is written $\Delta t_{AB}$.

With the knowledge of the refractive index n and the angle subtended by detector A from horizontal, $\theta_{D}$, the size of the particle can be written as:

(9) $$\begin{array}{c} D=\frac{2s\Delta t_{AB}\sqrt{1+n^{2}-2n\;cos\left(\frac{\theta_{D}}{2}\right)}}{\Delta t_{A}n\sin\left(\frac{\theta_{D}}{2}\right)}\; \end{array}$$

This equation is only valid for liquid hydrometeors.

### 4.3. Radar Disdrometers

Microwave or radar disdrometers, are low-power, small-size Doppler radars, vertically looking (without scanning capabilities) that exploit the Doppler effect due to falling drops to derive the spectrum of precipitating particle size. Continuous Wave (CW) radars are being widely adopted by National Weather Services and researchers thanks to their relative low cost and compact footprint.

They operate by measuring the power of the backscattered signal (*P_r_*) and its Doppler shift to estimate the Particle Size Distribution (PSD) by detecting hydrometeors passing through their sensing volume, as described by [3] These instruments are composed of two antennas, one that transmits at a constant frequency (*λ*) and one that receives the return signal.

A falling drop, moving vertically toward the instrument and crossing the equi-phase surfaces, produces a return signal with a power (*P_r_*) that can be expressed as follows

(10) $$P_{r}=\frac{P_{t}\cdot L_{w}\cdot G_{t}\left(R1\right)\cdot G_{r}\left(R2\right)\cdot \lambda^{2}\cdot \sigma }{64\pi^{3}{\left|R1\right|}^{2}{\left|R2\right|}^{2}}\;$$

This is a function of the transmitted power (*Pt*), the transmission loss (*L_w_*) due to the presence of water on the radomes, the distances between the emitting antenna and the falling drop (*R*1), and between the drop and the receiving antenna (*R*2), the antennas’ respective gains (*G_t_*(*R*1) and *G_r_*(*R*2)), the emitted frequency (*λ*) and the scattering cross-section (*σ*).

As the scattering cross-section can be numerically computed using the Rayleigh scattering or the Mie theory, depending on the instrument operating frequency, and the drop size D, the equation above can be written as

(11) D=[96λ2·Pr·|R1|2 |R2|2π2Pt·Lw·Gt(R1)·Gr(R2)(n2+2n2−1)2]16 

where *n* is the complex refractive index, a function of temperature and λ.

### 4.4. Contribution of Discretization

The resolution of the instruments is defined by the number of classes used to sort the raindrops. The discrete values of the particle size distribution (PSD) given by the instrument have an influence on the computed rainfall intensity. Taking the mean value of each bin as the particle size will introduce one part of the uncertainty linked to the discretization of the measured diameter.

The uncertainty on $D_{ref}$ will also have an influence here: the classification of the size of the drop will depend on the value of $D_{ref}$, and since it is a pdf, it can represent several bins in the discretization.

In order to evaluate the uncertainty value, we can see that it will depend on the drop generator used, but also on the instrument under test, since the classes are determined by the manufacturer. A straightforward approach is to make the convolution between the pdf of $D_{ref}$ and the classes of the instrument.

## 5. Uncertainty Block TRANS2 and IND2

For impact disdrometers, where we do not have access to the PSD used by the device, and to understand the complete process of all the instruments studied here, we have to describe the second transformation block and the final reading, the rain intensity, on the device. The second stage of our uncertainty model is focused on the following concepts.

### 5.1. Filtering and Rain Intensity Model

Each manufacturer has implemented its own algorithm to filter out events that are not related to actual rain drops, and based on the PSD/PVSD, to compute the corresponding rainfall intensity.

Since this information is not accessible, we cannot quantify the uncertainty linked to this treatment, but when comparing the rain intensity (RI) that we compute from the PVSD given by the device and its indication of RI for the same data, we can observe differences. The signal processing made in TRANS2 contribute to the total uncertainty on the RI expressed by the device, since the base assumptions made when implementing the filtering of abnormal data and the processing of the data might not be met during the measurement.

### 5.2. Quantities of Influence

The evaluation of influence parameters, affecting the behavior of non-catching precipitation instruments, is needed to perform a reliable quantification of the associated uncertainty and for the assessment of the accuracy of rain measurements, and will be reflected on the reading of the instrument (IND2).

The influence of air temperature and humidity on different models of non-catching precipitation instruments were performed at CEM, under controlled environmental conditions in a 2 m high climate chamber. This activity was conducted with the drop generator designed and manufactured by SMD and previously characterized in terms of its sensitivity to air temperature and humidity variations. This previous study demonstrates that the drop generator sensitivity is low enough to be used for the characterization of non-catching instruments. In addition to the drop generator, the following instrumentation was involved: calibrated thermometers (Pt-100), an anemometer, hygrometer and a calibrated scale (*U*(*k* = 2) = 0.10 mg).

A special configuration of the position of the drop generator and the instrument under study was defined to minimize the measurements’ dependence on the air currents, generated inside the climate chamber with the aim of reaching isothermal conditions. Despite this fact, special attention was paid to fit each generated drop into the sensing area of the rain instrument, in each of the measurements.

**Figure 6:**
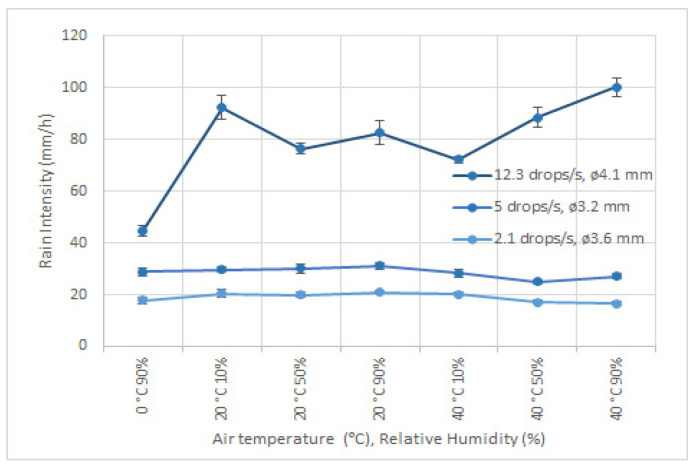
Influence of air temperature and humidity conditions on rain intensity measurement of one impact disdrometer, for different constant drop generator characteristics. On the top curve, the drop generator is set to produce series of 4.1 mm drops at 12.3 drops/s, in the middle figure, the values are 3.2 mm drops at 5 drops/s and the bottom figure show the results for values of 3.6 mm drops at 2.1 drops/s.

Figure 6 shows the mean values over 10 min of measurements for one impact disdrometer reading under different air temperature and humidity conditions and for different constant drop generator specifications, to produce for each curve a stable rain intensity. The vertical bars are the standard deviation of all measurements taken in the time interval. Results suggest that the dependence of impact disdrometers on air temperature and humidity is stronger for high rain intensities and at low temperature.

**Figure 7:**
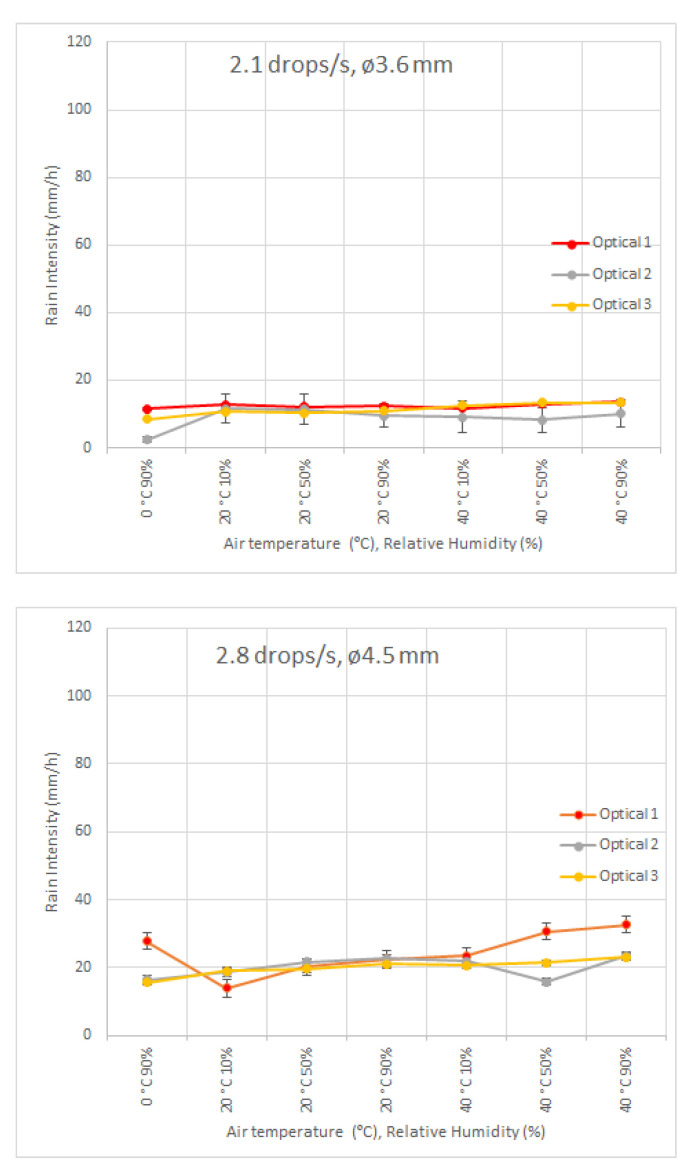

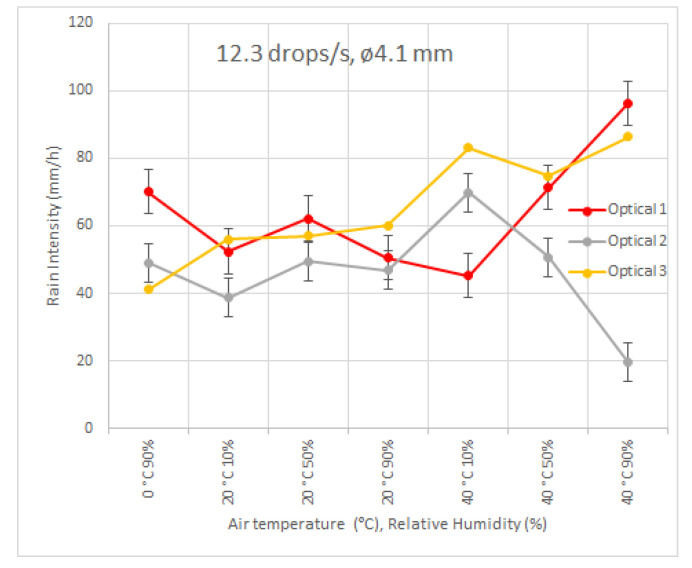
Influence of air temperature and humidity conditions on rain intensity measurement of three different models of optical transmission disdrometers, for different drop generator characteristics. On the top figure, the drop generator is set to produce 3.6 mm diameter drops at 2.1 drops/s, in the middle figure, the values are 4.5 mm drops at 2.8 drops/s and the bottom figure show the results for values of 4.1 mm drops at 12.3 drops/s.

In addition, three different models of optical disdrometers were also studied and the results are summarized in Figure 7. The vertical bars are, as for Figure 6, the standard deviation of the all the measurements performed in a 10 min time interval at each environmental condition.

As in the case of impact disdrometers, the influence of air temperature and humidity is more evident for high intensity rains and for extreme conditions of air temperature and humidity.

## 6. Conclusions

A model for the calibration uncertainty of non-catching precipitation gauges was proposed and detailed. The approach detailed in this paper is based on separating uncertainty components into blocks. This gives a flexibility on designing a calibration procedure by combining different blocks (i.e., changing the drop generator or the type of instrument). 

The first block element for this model is grouping the sources of uncertainties related to the reference droplet diameter. To further investigate and quantify the components three drop generators, based on different principles, were designed. For each drop generator the paper presents the characterization results and therefore the generated droplet sizes with the corresponding uncertainties.

The second block is related to the disdrometer itself and to the physical principles of operation. Impact, radar or optical disdrometers will show specific uncertainties that can be further explored starting from the specific model equations. One common component is related to the discretization, this latter being not only principle dependent but also manufacturer dependent. The block related to instruments will also include the influence of environmental parameters. Results from a measurement campaign performed with two types of non-catching rain gauges and a rain generator into a climate chamber give a first insight on the magnitude of the influences related to air temperature and humidity. For both types of instruments, higher effects are recorded at higher rain intensity and lower temperatures.

The third block in the model will group the effects related to the signal treatment. The information on filtering and calculation algorithms is not publicly available but we observed significant differences in rain intensity values indicated by the instrument and those computed using raw data when available. The main filtering component is related to the final velocity of the falling drop and this might be a major concern when developing an easy to implement calibration procedure. The drop generator needs to either eject drops with an initial velocity or be placed at a height that will ensure reaching the final velocity by free fall, this latter option being not easily obtained in normal laboratory buildings.

A current limitation of the proposed approach is the adopted use of liquid water drops to perform calibration in the laboratory. Since non-catching instruments are also used to measure solid precipitation, calibration using crystals in various aggregation forms should be addressed as well. However, producing snowflakes, graupels or other solid particles with known characteristics (shape, density, fall velocity) is still a challenging task for laboratory equipment. Therefore, intercomparison campaigns in the field using properly calibrated instruments seem to be, at present, the most suitable way ahead and are highly recommended.

The present paper is focused on uncertainty related to the calibration in laboratory conditions (calibration uncertainties). Additional influences are associated with the measurement of precipitation by means of non-catching instruments operating in the field (measurement uncertainties). Environmental sources of bias are documented in the literature, especially due to the wind and turbulence developed due to the bluff-body behavior of the instrument itself (see e.g., [20,21]). Further work is needed to encompass both instrumental and environmental sources of bias and uncertainty and to provide an overall uncertainty budget for non-catching precipitation measuring instruments. Additional information is also required from the manufacturers to make the link between the calibrated drop size and velocity and the resulting rain intensity indicated by the devices.

## Figures and Tables

**Table 1:** The nozzles/tubes installed in the individual drop-generator units and the expected resulting drop diameter.

Position	Nozzle/Tube	Drop Diameter (mm)
1	0.1 mm nozzle	≤0.2 mm
2	0.2 mm nozzle	0.3 mm
3	0.4 mm nozzle	0.5 mm
4	0.8 mm nozzle	0.9 mm
5	1.0 mm nozzle	1.1 mm
6	1.3 mm tube/needle	2.2–3.3 mm
7	1.8 mm tube/needle	≤4.0 mm
8	Special nozzle	7 mm

**Table 2:** Validation of the photogrammetric device.

Test n.	Number of Drops	Avg D (mm) from the Camera	Avg D (mm) from the Balance	Difference (mm)	Fraction of the Terminal Velocity (%)
1	21	1.538 ± 0.058	1.551 ± 0.344	−0.013	72
2	43	3.072 ± 0.072	3.026 ± 0.090	0.046	54
3	50	5.129 ± 0.276	4.978 ± 0.111	0.152	44

**Table 3:** Characterization of the DG2 in terms of the drop diameter.

Test n.	Number of Drops	Avg D (mm)	Std Dev D (mm)	Max D (mm)	Min D (mm)	Theoretical Fall Velocity Average Drop (m/s)	Fraction of the Terminal Velocity (%)
1	50	3.42	0.15	3.77	3.08	4.47	50.8
2	50	1.77	0.05	1.98	1.70	4.06	68.4
3	50	1.12	0.04	1.25	1.06	3.50	81.4
4	50	0.85	0.01	0.89	0.83	3.02	91.0

**Table 4:** Characterization of the DG2 in terms of the drop fall velocity.

Test n.	Number of Drops	Avg V (m/s)	Std Dev V (m/s)	Max V (m/s)	Min V (m/s)	Theoretical Fall Velocity Average Drop (m/s)	Fraction of the Terminal Velocity (%)
1	50	4.36	0.12	4.56	3.98	4.47	50.8
2	50	3.79	0.10	4.08	3.65	4.06	68.4
3	50	3.29	0.05	3.19	4.54	3.50	81.4
4	50	2.72	0.04	2.81	2.65	3.02	91.0

**Table 5:** Characterization of the different nozzles available for the DG3. The drop frequency was measured, and the drop weight is the mean weight of at least three different measurements. Drop size is calculated from drop weight assuming spherical shape.

Nozzle	Drop Frequency (Drops/s)	Drop Weight (mg)	Drop Size (mm)	Probability Distribution
A	3.4	9.5 ± 0.09	2.6 ± 0.02	Gaussian
B	2.1	23.5 ± 0.21	3.6 ± 0.02	Gaussian
D	2.8	44.5 ± 0.13	4.4 ± 0.01	Gaussian
E	2.4	61.7 ± 1.56	4.9 ± 0.08	Gaussian
F	5.0	8.1 ± 0.08	2.5 ± 0.02	Gaussian

## Data Availability

Data for the UNIGE drop generator (DG2) related to Table 2, Table 3 and Table 4 can be found at https://doi.org/10.5281/zenodo.6958498. Data for the SMD drop generator (DG3) related to Table 5 can be found at https://doi.org/10.5281/zenodo.6962651.
